# Acute metabolite responses to swimming exercise of different intensities in highly trained male and female swimmers

**DOI:** 10.14814/phy2.70532

**Published:** 2025-08-26

**Authors:** Andew D. Govus, Lachlan J. G. Mitchell, Chloe D. Goldsmith, Katie E. McGibbon, David B. Pyne, Maria Kozlovskaia, Nathan G. Lawler

**Affiliations:** ^1^ Discipline of Sport and Exercise Science La Trobe University Bundoora Victoria Australia; ^2^ Victorian Institute of Sport Albert Park Victoria Australia; ^3^ School of Science, Paramatta South Campus University of Western Sydney Paramatta, Sydney New South Wales Australia; ^4^ Performance Science Unit Queensland Academy of Sport Nathan Queensland Australia; ^5^ Research Institute Sport and Exercise University of Canberra Canberra Australian Capital Territory Australia; ^6^ Centre for Computational and Systems Medicine, Health Futures Institute Murdoch University Perth Western Australia Australia

**Keywords:** biochemistry, energetics, metabolomics, phenotype

## Abstract

We investigated metabolite responses to different swimming intensities in 16 highly trained swimmers (9 males, 7 females, aged 16–24 years). After determining critical swimming speed (CS) with a 12 × 25 m maximal effort test, participants completed three swimming trials at moderate (below CS), heavy (at CS), and severe (above CS) intensities on separate days. Capillary blood samples (1 mL) were collected before and after each trial for metabolite profiling via mass spectrometry. Orthogonal partial least squares analysis (OPLS‐DA) revealed distinct metabolite changes between moderate and severe intensity trials [R^2^X (cum): 0.56; R^2^Y(cum): 0.95, pR^2^Y: 0.02, pQ^2^: 0.02]. Free fatty acids (FFAs) 18:0, 20:0, 20:4, and 22:4 showed higher log_2_ fold changes (log_2_FC) after moderate compared to heavy and severe trials (all *p* < 0.01). Plasma lactate, pyruvate, alanine, and HDL‐4 cholesterol concentrations had greater log_2_FC after heavy and severe trials than moderate (all *p* < 0.01). Males tended to show lower log_2_FC in FFA than females in the severe trial, though this was not significant. These findings demonstrate that swimming intensity influences metabolite profiles, with reduced lipid metabolism and increased TCA cycle activity as intensity rises. A 1 mL capillary blood sample can effectively capture these metabolic shifts.

## 1 INTRODUCTION

Swimmers train at different intensities during a weekly training cycle to enhance their fitness and performance for their chosen competitive swimming event(s). Swimming training sessions typically include exercise performed above and below their critical swimming speed, that is, the swimming speed that defines the boundary between the heavy and severe exercise intensity domains (Wakayoshi et al., 1992). The physiological premise of this approach is to preferentially develop the swimmer's ability to supply skeletal muscle with adenosine triphosphate (ATP) via oxidative phosphorylation, glycolysis, and high‐energy phosphate pathways (Hargreaves & Spriet, 2020). Depending upon a swimmer's target event (sprint: 50–100 m, middle distance: 200–400 m, and distance events: 800–1500 m) and the current phase of the season, the volume of work performed below or above a swimmer's critical swimming speed in a training programme will target oxidative, glycolytic, or phosphagen energy pathways (Hellard et al., 2019). Sports scientists and swimming coaches monitor several internal (heart rates and blood lactate) and external load (e.g., lap times, stroke rates, and stroke counts) measures during and/or after each swimming session to understand how swimmers are physiologically responding and adapting to training. However, traditional measures of external and internal training load do not provide a comprehensive overview of the molecular response stimulated by each swimming session. A more detailed understanding of the acute molecular responses to swimming training will help coaches and sport scientists better individualize training prescription and periodisation based on a swimmer's individual molecular profile (Pla et al., 2021).

Molecular profiling methods leverage analytical and biological chemistry, particularly through liquid chromatography‐mass spectrometry (LC–MS) and nuclear magnetic resonance (NMR) spectrometry, coupled with computational biology, to comprehensively map molecular pathways involved in cellular and systemic functions from very small quantities of blood (~20–50 μL) (Nicholson, 2006). This approach can uncover diverse biological layers such as the metabolome, which is a complex profile of metabolites involved in cellular energy production, signaling, and biosynthetic pathways. Quantitatively and qualitatively analyzing the metabolome using LC–MS and NMR provides precise insights into how environmental factors including diet, exercise, and stress influence cellular processes and overall health. Collectively, this work should provide valuable insights into an athlete's molecular responses to training, exercise recovery, and adaptation (Belhaj et al., 2021).

Metabolic phenotyping has so far shed light on the post‐exercise metabolic responses to exercise performed at different intensities across multiple metabolic pathways. During prolonged, low or moderate intensity (<75% V̇O_2peak_) endurance exercise, long and medium side chain fatty acids (LCFA and MCFAs) are mobilized from triacylglycerols (TAGS) and diacylglycerols (DAGS) located in skeletal muscle near mitochondria and oxidized to form ATP via beta oxidation (Gemmink et al., 2020). Some circulating plasma free fatty acids (FFAs) are also oxidized to supply ATP during exercise. Plasma FFA oxidation is lower in endurance‐trained athletes compared to untrained nonobese individuals as athletes have a greater overall intramyocellular lipid content (Amati et al., 2011), with lipid droplets organized as smaller and easier to access units that are more decorated by perilipin 2 and 5 (Gemmink et al., 2021). Following prolonged low‐intensity exercise, TCA intermediates (e.g., succinate, citrate, fumarate, and malate) may also “spill over” from skeletal muscle and liver into the plasma. In comparison, exercise training sessions performed in the heavy or severe exercise intensity domains (typically >75% V̇O_2peak_) increase the abundance of metabolites involved in the glycolysis pathway (e.g., lactate and pyruvate), TCA cycle (e.g., citrate, succinate, fumarate, and malate) (Peake et al., 2014), glucose‐alanine cycle (i.e., alanine), purine metabolism pathways (e.g., hypoxanthine, xanthine, and uric acid) (Zieliński & Kusy, 2015), and to a smaller extent lipolysis pathways [e.g., medium chain fatty acids (MCFAs), long chain fatty acids (LCFAs), and very low density lipoproteins (VDLs)] (Danaher et al., 2016; Peake et al., 2014; San‐Millán et al., 2020).

The metabolic responses to exercise can be divided into immediate (<1 h), early (1–4 h), and late (>4 h) phases (Lundsgaard et al., 2020). Deep molecular profiling of the changes in gene transcripts, proteins, metabolites, and complex lipids after a graded exercise test performed by 36 untrained males aged 40–70 years revealed an increase in inflammatory cytokines, markers of oxidative stress, FA oxidation, and lipid signaling within 2 min after exercise, followed by an increase in markers of tissue repair within 15 min after exercise (Contrepois et al., 2020). During early and late exercise recovery, LCFAs, MCFAs, and VLDL concentrations progressively decrease as FAs are used to replenish muscle glycogen, and amino acids either increase or decrease in concentration depending on whether they are used for glycogen resynthesis, repairing damaged tissues, or detoxifying ammonia. However, exercise intensity, duration, pre‐exercise fuelling, training history, sex, age, and the timing of sample collection after exercise can affect the acute molecular response to exercise and alter the metabolite composition of the plasma at the time of collection (Schranner et al., 2020).

Metabolic phenotype analysis in high‐performance swimmers would provide useful and novel information about the molecular profile differences in response to swimming trials performed at different exercise intensities. Techniques such as metabolic profiling explore the dynamic interaction between an individual's metabolism and their environment, which includes their training program, dietary and sleep habits, and life stressors. Developing molecular profiling tools to monitor athletes will provide sports scientists and coaches with a more detailed understanding of the molecular changes induced by exercise. This work can also provide a more comprehensive insight into how nutrition and recovery methods affect exercise training response than can be achieved with traditional athlete profiling tools. This study explored the acute changes in plasma metabolite profiles from rest in highly trained male and female swimmers following three swimming trials performed within the moderate, heavy, and severe exercise intensity domains.

## 2 METHODS

### 2.1 Study design

**FIGURE 1:**
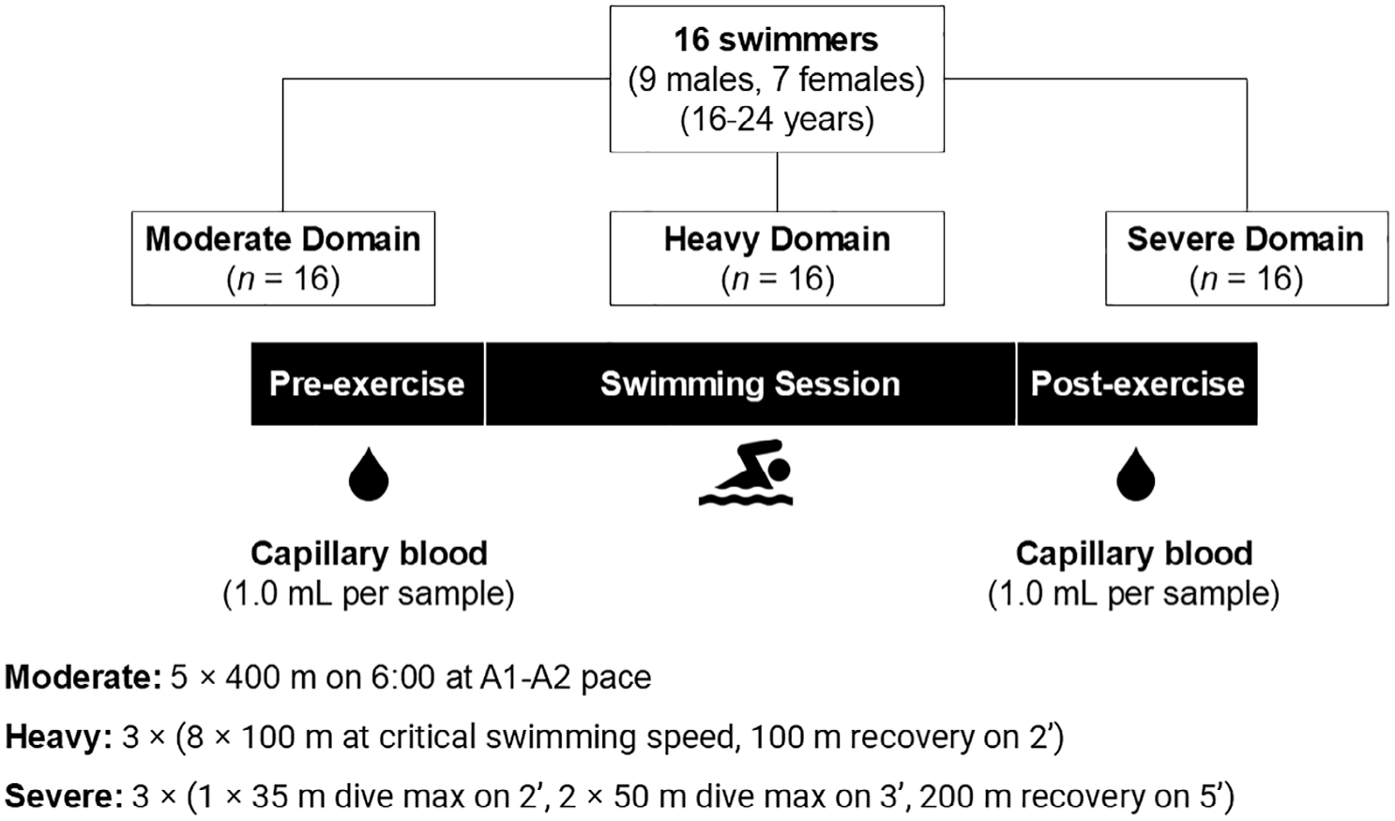
Study design schematic. Participants performed a 12 × 25 m critical speed test approximately 1 week before the test week. During the testing week, participants performed three standardized swimming trials in the moderate, heavy, and severe exercise intensity domains, which were scheduled 1 day apart. To profile participants' metabolic and epigenetic responses to exercise, a capillary blood sample (1.0 mL whole blood) was collected from a fingertip after 5 min of hand warming before and immediately after each standardized swimming trial.

Ethical approval for this study was granted by the La Trobe University Human Research Ethics Committee (ethics approval number: HEC21001). This study employed a repeated measures design involving four separate swimming trials in a 25‐m pool. Sixteen highly trained (Tier 3) (McKay et al., 2021) swimmers performed a 12 × 25 m critical swimming speed test approximately 1 week before a series of three separate swimming trials: one in the moderate, heavy, and severe exercise intensity domains, scheduled 1 day apart in a randomized and counterbalanced order. To profile participants' metabolic and epigenetic responses to exercise, a capillary blood sample (1.0 mL whole blood) was collected from their fingertip after 5 min of hand warming before and immediately after each swimming trial and analyzed to determine changes in the metabolomic and epigenetic profile (data reported elsewhere) (Figure 1).

### 2.2 Participants

A total of 16 participants (9 males, 7 females, age: 16–24 years) were recruited from two local swimming squads within Queensland, Australia and provided their written informed consent to be involved in the study. At the time of data collection, participants were ranked within the top 200 in their event within Australia (World Aquatics Points 2022–2023: mean: 723, standard deviation: 53). All participants were either sprint (50 m, 100 m) or middle‐distance swimmers (200 m, 400 m). Two swimmers specialized in breaststroke, two in backstroke, one in butterfly, with the rest swimming mostly freestyle (front crawl). Participants completed an online pre‐exercise medical screening questionnaire (ESSA Adult Pre‐Exercise Screening Form), were nonsmokers, free of metabolic disorders, and not taking any medication (other than oral iron supplements and/or asthma medication with an appropriate therapeutic use exemption). Female participants taking hormonal contraceptives capable of altering the endogenous hormonal milieu for at least 3 months before testing were not excluded from the study.

### 2.3 12 × 25 m critical swimming speed test

A 12 × 25 m swimming test was conducted in a 25 m pool approximately 1 week before the main testing period to determine swimmers' critical swimming speed (CS) and exercise intensity domains (Mitchell et al., 2018, 2019). Before the test, participants were instructed to abstain from intensive exercise for 24 h and complete a self‐selected race‐specific swimming warm‐up of ~1000 m followed by four to 5 min of passive rest. Thereafter, participants completed 12 × 25 m consecutive maximal swimming efforts in their specialist stroke from a push start, each separated by 5 s of passive rest. Butterfly and medley swimmers were allowed to complete the test using front crawl (freestyle) unless they had a secondary specialist stroke.

A maximum of two underwater butterfly kicks was permitted at the beginning of each effort for backstroke and front crawl, with breaststroke swimmers allowed to complete an underwater pullout. To offset the potential effects of pacing, swimmers were instructed to swim each 25 m interval with maximal effort and leave nothing in reserve for the rest of the test. The time for each 25 m segment and stroke rate for each three swimming stroke cycles was measured using a handheld stopwatch (Seiko, Tokyo, Japan). Peak blood lactate was sampled (5 μL) from the earlobe every 2 min (Lactate Pro 2, Arkray Inc., Kyoto, Japan), beginning 1 min after the test and concluding when the blood lactate concentration stabilized or decreased.

Peak swimming speed, critical speed (CS), and drop off (%) were calculated from the results of the 12 × 25 m test (Mitchell et al., 2019). Peak speed was calculated as the mean speed of the first 25 m effort. The CS was calculated as the mean speed of the slowest two 25 m efforts within the final four 25 m efforts. Drop off (%) was calculated as the percentage difference between the peak speed and the CS. D′ represents the integration of the Speed‐Time model, based upon Equations 1 and 2 below:

(1) $$S_{t}=ae^{\mathrm{bt}}+c$$

(2) $$D^{\prime }=\left(\left(t_{1}\times S_{1}\right)-\left(\mathrm{CS}\times t_{1}\right)\right)+\left(\left(\int_{t12}^{t_{1}}ae^{\mathrm{bt}}+c-\mathrm{CS}\right)\right)$$

Where: *S*_*t*_ = speed at time (*t*), *e* = Euler's number, *a, b, c* are weighting factors, *t*_1_ = total time (s) at the end of the twelfth 25 m effort, and CS = critical speed (m/s) calculated as the slowest of the two 25 m efforts in the last four efforts of the test.

### 2.4 Swimming trials

Swimming trials were conducted in a 25 m pool. Each of the three swimming trials was performed at the same time of day (~06:30–07:00) during one training week to minimize the effect of circadian variation on the plasma metabolome (Dallmann et al., 2012). Swimmers were asked to abstain from caffeine and alcohol for 24 h before each swimming trial and replicate their food intake (breakfast) before each trial.

After a standardized warm up (200 m easy swim, followed by 6 × 50 m efforts as 25 m pace/25 m recovery), swimmers performed in a randomized and counterbalanced order the three swimming trials in the moderate, heavy, and severe intensity domains scheduled 1 day apart (Table 1). Capillary blood lactate samples (5 μL) were collected from swimmers' earlobe at the using an automated, handheld blood lactate analyser (Lactate Pro 2, Akray Inc., Kyoto, Japan) conclusion of the moderate domain session (one sample total) and after each main set during the heavy and severe intensity domain sessions (three samples per swimmer for each session).

**TABLE 1:** Standardized swimming trials performed in the moderate, heavy, and severe exercise intensity domains. A1: Aerobic intensity, zone 1; A2: Aerobic intensity, zone 2 (Mujika et al., 1995).

Exercise intensity domain	Trial
Moderate	5 × 400 m on a 6‐min time cycle at A1/A2 swimming speed
Heavy	3 × (8 × 100 m holding critical speed, 100 m recovery on a 2‐min time cycle)
Severe	3 × (1 × 35 m dive max on a 2‐min time cycle, 2 × 50 m dive max on 3‐min time cycle, 200 m recovery on 5‐min time cycle)

### 2.5 Capillary blood collection, storage, and transport protocols

Capillary whole blood (1.0 mL) from a fingertip was collected into a lithium heparin collection tube (Minicollect, Greiner Vacuette, USA) before and immediately after (within ~15 min) each swimming trial, following 5 min of hand warming to enhance local blood flow. The capillary blood samples were then centrifuged for 15 min at 1500 × *g* to separate the plasma from the red blood cells. Subsequently, 100 μL (0.1 mL) of plasma was pipetted into a 1.5 mL Eppendorf tube for transport. Both plasma and whole blood samples were stored immediately on ice and then at −80°C within 2 h post‐collection. The samples were transported on dry ice via a medical courier for metabolic and epigenetic profiling (data reported elsewhere).

### 2.6 Metabolomics profiling

Blood plasma was analyzed using a combination of quantitative nuclear magnetic resonance spectroscopy (NMR) and targeted liquid chromatography mass spectrometry (MS) platforms to give broad lipoprotein, lipidomic, and amino acid coverage.

#### 2.6.1 ^1^H nuclear magnetic resonance (NMR) spectrometry

^1^H NMR Spectrometry was performed using established methods (Dona et al., 2014), as follows: plasma samples were thawed at room temperature for 1 h before preparation for analysis. Each sample volume of 90 μL of plasma was mixed with 90 μL of phosphate buffer (75 mM Na_2_HPO_4_, 2 mM NaN3, 4.6 mM sodium trimethylsilyl propionate‐[2,2,3,3–2H_4_] (TSP) in D20, pH 7.4 ± 0.1) and transferred to a 3 mm SampleJet NMR tube, sealed with a POM ball added to the cap. The NMR spectroscopic analyses were performed on a 600 MHz Bruker Avance III HD spectrometer equipped with a 5 mm BBI probe fitted with a Bruker SampleJet robot cooling system set to 5°C. A full quantitative calibration was completed before analysis using previously published methods (Dona et al., 2014). All experimental data were acquired using the Bruker In Vitro Diagnostic research (IVDr) methods. For each sample, two experiments were completed in automation mode, amounting to a total of 12.5 min acquisition time per sample: a standard 1D experiment with solvent pre‐saturation (128 scans using a mixing time of 0.01 s and relaxation delay of 4 s, 96K data points, spectral width of 30 ppm, line broadening of 0.3 Hz, zero‐filled to 128 K), and a JEDI‐PGPE experiment (256 scans, relaxation delay of 1 s, 96k data points, spectral width of 30 ppm, line broadening of 0.3 Hz, zero‐filled to 128 K) was conducted. Data were processed in automation using Bruker Topspin 3.6.3 and ICON NMR to achieve phasing and baseline correction. A total of 112 lipoprotein parameters for each sample were generated using the Bruker IVDr Lipoprotein Subclass Analysis (B.I.LISA) method. This panel was obtained by mathematically interrogating and quantifying the −CH_2_ (δ = 1.25) and −CH_3_ (δ = 0.80) peaks of the 1D spectrum after normalization to the Bruker QuantRef manager within Topspin using a PLS‐2 regression model.

#### 2.6.2 Liquid chromatography (LC)‐mass spectrometry (MS)

Targeted lipidomic analysis was conducted where samples were extracted and prepared following our in‐house published protocols (Ryan et al., 2023) with some modifications to internal standard mixtures. Briefly, plasma samples were thawed at 4°C, and 10 μL were transferred to 96‐well plates. Samples were vortex mixed with 90 μL of propan‐2‐ol containing stable isotopically labeled standards, which were diluted to achieve a final concentration of 1 in 500 (Avanti Polar Lipids UltimateSPLASH™ ONE (product number: 330820L), SphingoSPLASHTM I (product number: 330734W) and 0.01 μg/mL MG 18:1‐d7 (product number: 791646C) and 0.005 μg/mL oleic acid‐d9 (product number: 861809O) (Sigma‐Aldrich, North Ryde, NSW, Australia), arachidonic acid‐d5 (product number: 9000477), linoleic acid‐d11 (product number: 9002193), palmitic acid‐d5 (product number: 30557), and stearic acid‐d4 (product number: 30549) from Cayman Chemical (Sapphire Bioscience, Redfern, NSW, Australia)). Samples were centrifuged at 14,000 *g* for 10 min, and the supernatant was transferred to a fresh 96‐well plate. The LC–MS method was run on a SCIEX ExionLC and QTRAP 6500+ with electrospray ionization using polarity switching. Data were acquired using SCIEX Analyst (v1.7.1). Separation was performed using a Waters Acquity BEH C18 1.7 μm, 2.1 × 100 mm column (Waters Corp., MA, USA) at 60°C. The injection volume was 5 μL, and samples were kept in the autosampler at 10°C. Time‐scheduled multiple reaction monitoring (MRM) was used for data acquisition of 1230 transitions (including 1163 lipid species and 67 internal standards).

### 2.7 Metabolomics data processing and statistical analyses

#### 2.7.1 Data cleaning

##### Lipid analysis

Independent plasma pool (QC sample) was prepared as per the samples, and aliquots then injected following each block of 10 experimental samples throughout the analytical sequence, which were used to assess analytical precision. Obtained raw files were pre‐processed using SkylineMS (Adams et al., 2020). Feature filtering, RSD QC > 30%, and feature intensity threshold filtering <5000 in >50% of the QCs were applied, and metabolites were removed from further statistical analysis if they did not meet this analytical precision. Statistical analyses were performed using R version 4.2.3 (R Core Development Team, 2023). Metabolites with >70% missing data were removed from the dataset. For the remaining data, missing data were imputed using a random forest algorithm from the *missForest* package (Stekhoven & Bühlmann, 2012).

#### 2.7.2 Orthogonal partial least squares discriminant analysis (OPLS‐DA)

**FIGURE 2:**
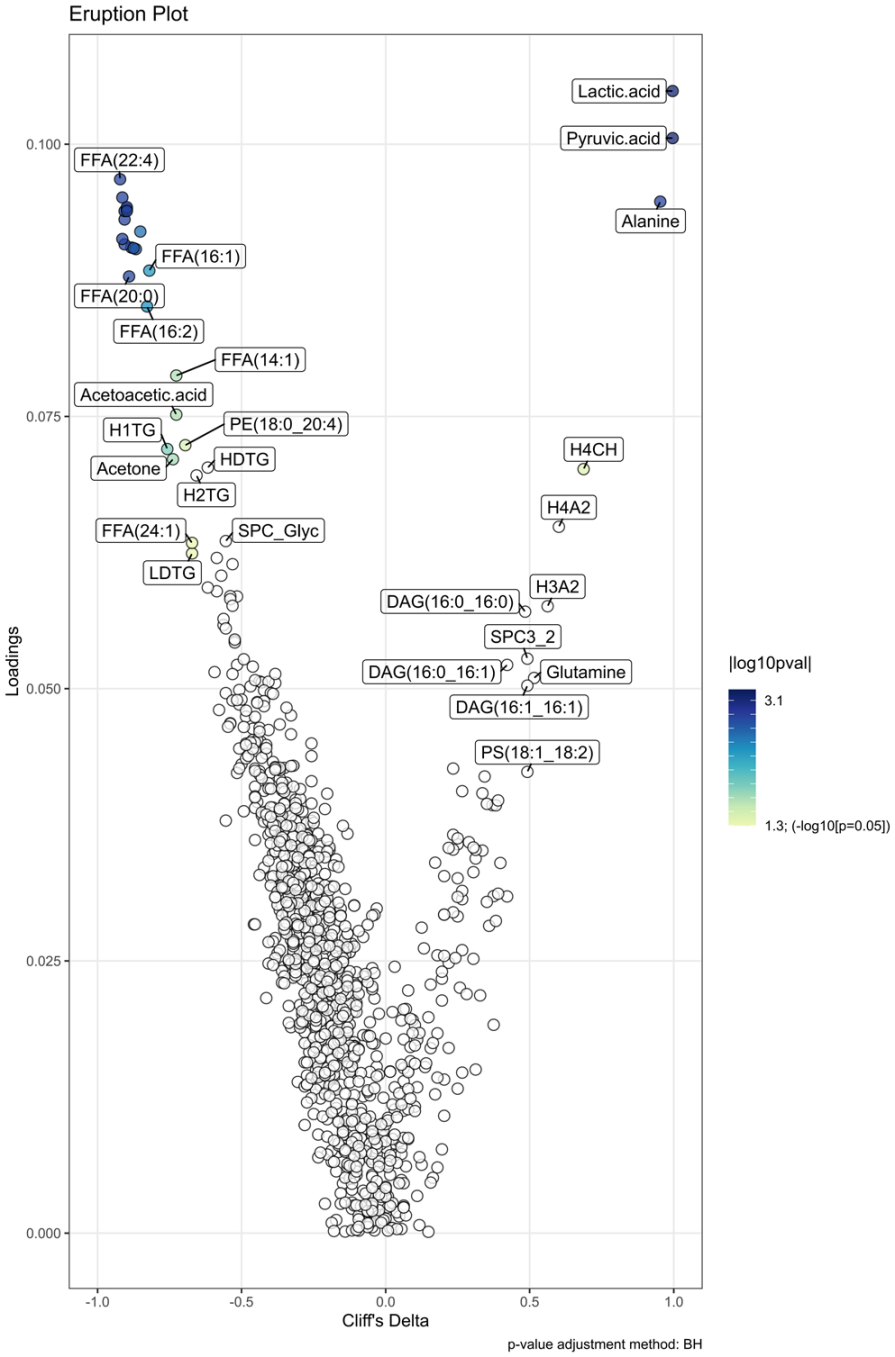
Eruption plot displaying Cliff's delta for key metabolites discriminating the pre‐ and post‐exercise time points within the moderate and severe intensity domain swimming trials.

An OPLS‐DA was fitted using the *mva.plots* package (https://github.com/phenological/mva‐plots) to determine a set of metabolites that best discriminated between the log_2_ fold change for each exercise condition (3 labels: moderate, heavy, and severe). Data were auto‐scaled and mean‐centered before analysis. The reliability of each OPLS‐DA was evaluated using 8‐fold cross validation and permuted 50 times to reduce overfitting with the R^2^Y (goodness of fit indicator) and Q^2^ (predictive ability indicator) >0.5. Metabolites with variable importance on projection (VIP) scores >3.0 and *p* < 0.05 for each trial were considered key features responsible for separating pre‐ and post‐exercise time points. An eruption plot (Figure 2) was employed to visualize the Cliff's delta effect size (Cliff, 1993) discriminating between the moderate and severe intensity domain swimming trials, with the −log_10_ *p* value colored to show statistical significance. A table detailing the overlapping metabolites between each swimming trial is available in the Appendix S1.

#### 2.7.3 Linear mixed model analyses

Separate linear mixed models were fit to each metabolite separately using the *nlme* (Pinheiro et al., 2011) or *lme4* packages (Bates et al., 2014). The model included fixed effects for time (categorical fixed effect; 2 levels: pre‐exercise, post‐exercise) and trial (categorical fixed effect; 3 levels: moderate, heavy, severe), a time × trial term, and controlled for sex (categorical fixed effect; 2 levels: male, female). A random intercept was fit for participant ID (categorical random effect: 14 levels). A heterogeneous variance structure involved modeling separate level 1 (within‐participant) variances for each swimming trial to account for potential heteroscedasticity (i.e., unequal variance) in metabolites abundances between swimming trials, where applicable (i.e., this process resulted in an improved model fit). Post hoc, pairwise comparisons were performed for significant interaction terms using the *emmeans* package (Lenth, 2018) applying the Benajmin‐Hochberg correction to the *p* values to control the false discovery rate (Benjamini & Hochberg, 1995). Bootstrapped 95% confidence intervals (95% CI) denote the standard error of the parameter estimates.

#### 2.7.4 Data Availability and Analysis Code

An anonymised version of the data used in the statistical analysis is available on FigShare to enable replication of the data analysis (https://doi.org/10.6084/m9.figshare.28502627). The R code to reproduce metabolomic analyses conducted in this study is available on the project's GitHub repository: https://github.com/NathanGLawler/Swimmer‐s‐Phenomics‐Project/tree/main/SMP1.

## 3 RESULTS

### 3.1 Swimming trials

The performance results of each swimming trial are summarized in Table 2.

**TABLE 2:** Average lap times (s), heart rate (beats/min), blood lactate concentrations (mmol/L) for male (*n* = 9) and female (*n* = 7) swimmers for trials performed in the moderate, heavy, and severe intensity domains. Lap times have been averaged for each set where sets were performed.

Variable	Moderate (5 × 400 m)				Heavy [3 × (8 × 100 m)]				Severe [3 × (1 × 35 m, 2 × 50 m)]							
	Females (400 m)		Males (400 m)		Females (200 m)		Males (200 m)		Females (35 m)		Males (35 m)		Females (50 m)		Males (50 m)	
	Mean	SD	Mean	SD	Mean	SD	Mean	SD	Mean	SD	Mean	SD	Mean	SD	Mean	SD
Time (s)	313.3	9.8	289.3	11.5	68.3	3.6	63.6	7.0	18.7	0.7	16.5	0.4	28.2	1.0	25	1.3
Heart rate (beats/min)	154	14	159	19	166	16	172	20								
Blood lactate (mmol/L)	1.3	0.3	1.8	0.8	3.6	1.6	4.2	2.0					9.6	5.3	11.0	3.0

### 3.2 Changes in metabolites pre‐ versus post‐exercise within swimming trials

Absolute quantifications were obtained for 112 lipoproteins and subfractions, and 23 small molecule metabolites from the targeted ^1^H NMR analysis. Semi‐quantification of 929 QC checked lipids was analyzed using a semi‐quantitative, targeted lipidomic LC–MS platform.

The OPLS‐DA analysis robustly discriminated between pre‐ and post‐exercise metabolite profiles in the moderate intensity domain (pR^2^Y = 0.02, pQ^2^: 0.02). The model explained 52% [R^2^X(cum) = 0.52] of the variance in predictor variables and achieved a strong fit, with 93% of the variance in the response variable explained [R^2^Y (cum) = 0.93]. The model demonstrated low predictive power on new data, with a Q^2^ (cum) of 0.46 and a root mean squared error of estimation (RSMSEE) of 0.14, reflecting a low error in the prediction response. In comparison, pre‐ and post‐exercise metabolite profiles could not be effectively separated using the first predictive component from the OPLS‐DA model in the heavy and severe domains.

### 3.3 Differences in log_2_ fold change (post‐exercise/pre‐exercise) of metabolites between swimming trials

**FIGURE 3:**
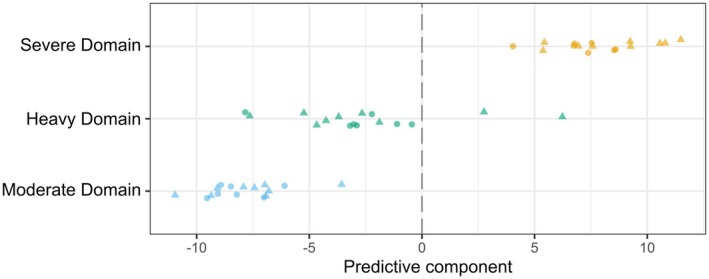
Heavy intensity domain metabolite responses projected over the results of the OPLS‐DA model built for the log_2_ fold changes in metabolites between the moderate and severe intensity domain swimming trials. A list of overlapping metabolites between each swimming trial are available in the Appendix S1.

**FIGURE 4:**
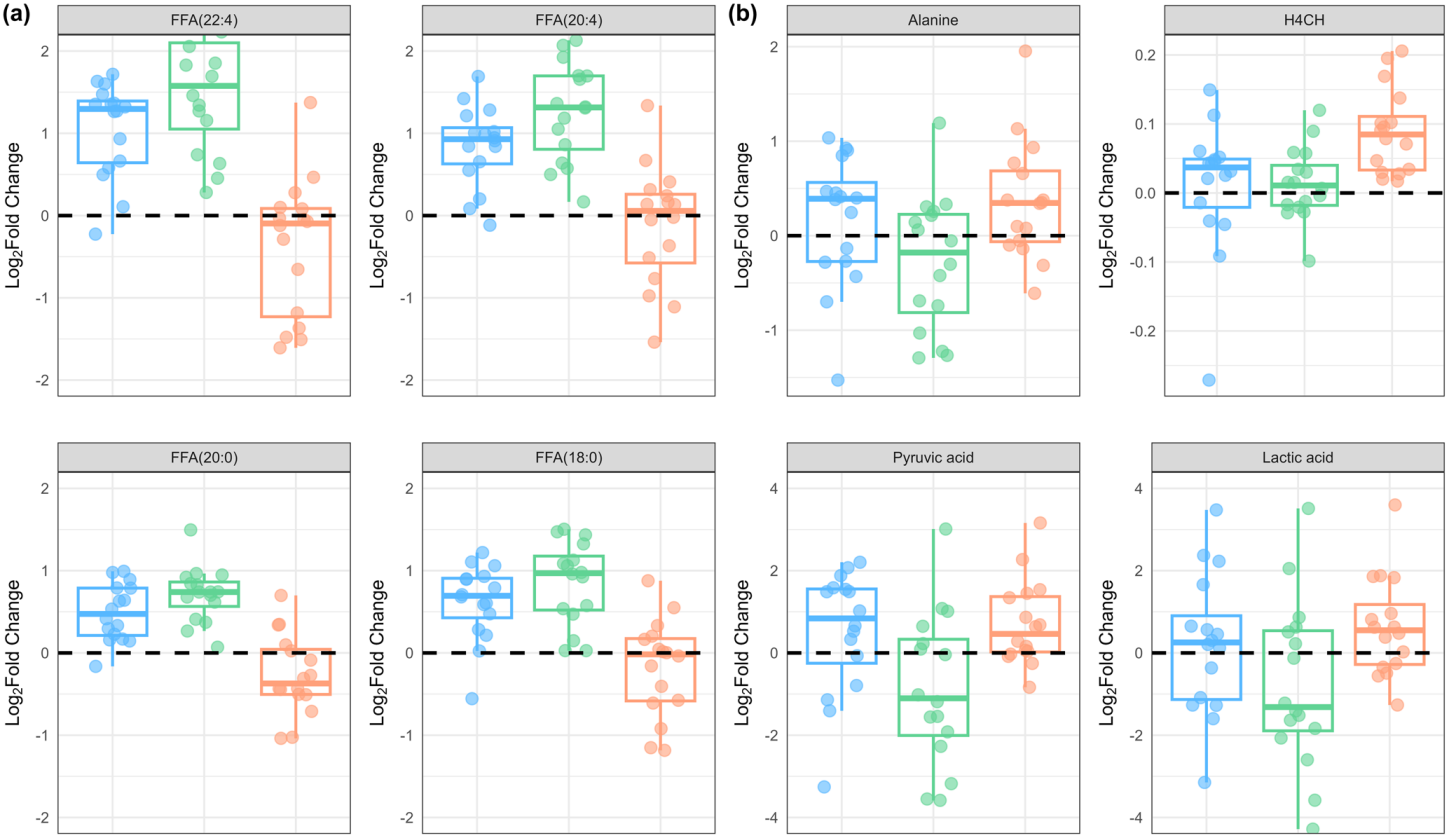
Log_2_ fold change (post‐exercise/pre‐exercise) for the top four metabolites from the OPLS‐DA model were followed up with a univariate linear mixed model. (a) Presents the top four metabolites that were higher in the moderate compared to the severe intensity domain swimming trial (all *p* < 0.01), and (b) the top four metabolites that were higher in the severe compared to the moderate swimming domain trial (all *p* < 0.001).

An orthogonal partial least squares discriminant analysis (OPLS‐DA) model of the log_2_ fold change values of metabolites was built to determine the metabolites (*n* = 1064 metabolites) that best discriminated between moderate and severe intensity domain trials. The model explained 56% of the variance in the predictor variables [R^2^X (cum): 0.56] and achieved a strong fit with 95% of the variance in the response variable explained by the model [R^2^Y(cum): 0.95]. The statistical significance of both the R^2^Y Q^2^ values (pR^2^Y: 0.02, pQ^2^: 0.02) indicates a robust differentiation between trials performed in the moderate and severe intensity domains (Figure 2). The metabolite profile from the heavy intensity domain trial was then projected on to the OPLS‐DA model built for the moderate versus severe domain trial to compare the metabolite profile between the moderate, heavy, and severe intensity domain trials (Figure 3). Univariate linear mixed models indicated that log_2_ fold concentrations tended to be higher following exercise in the moderate intensity domain than in the heavy and severe intensity domains for LCFAs including stearic acid (FFA 18:0) (moderate vs. heavy: *β* = 0.23, 95% CI: [0.09, 0.38], *p* = 0.002; moderate vs. severe: *β* = 1.03, 95% CI: [0.87, 1.19], *p* < 0.001), arachidic acid (FFA 20:0) (moderate vs. heavy: *β* = 0.22, 95% CI: [0.08, 0.36], *p* < 0.001; moderate vs. severe: *β* = 0.97, 95% CI: [0.82, −1.13]), arachidonic acid (FFA 20:4) (moderate vs. heavy: *β* = 0.42, 95% CI: [0.22, −0.60], *p* < 0.001; moderate vs. severe: *β* = 1.38, 95% CI: [1.18, 1.58], *p* < 0.001), and docosatetraenoic acid (FFA 22:4) (moderate vs. heavy: *β* = 0.42, 95% CI: [0.22, 0.42], *p* < 0.001; moderate vs. severe: *β* = 1.89, 95% CI: [1.68, 2.11], *p* < 0.001) (Figure 4a). The log_2_ fold change values tended to be higher after exercise in the severe compared to the moderate intensity domains for pyruvate (*β* = 1.58, 95% CI: [0.89, 2.20], *p* < 0.001), lactate (*β* = 1.42, 95% CI: [0.67, 2.15], *p* < 0.001), alanine (*β* = 0.64, 95% CI: [0.34, 0.94], *p* < 0.001), and H4CH (*β* = 0.08, 95% CI: [0.05, 0.11], *p* < 0.001) (Figure 4b).

## 4 DISCUSSION

We examined the metabolic phenotype from a 1.0 mL capillary finger prick blood sample associated with swimming trials performed within the moderate, heavy, and severe intensity domains in highly trained male and female swimmers. Metabolic phenotyping identified distinct metabolic responses in the log_2_ fold change (post/pre‐exercise) between swimming trials performed in different exercise intensities (as indicated by the OPLS‐DA model). The greatest differences were observed between swimming trials performance in the moderate and severe intensity domains, while the metabolic responses between the moderate and heavy domain trials were similar. The log_2_fold change in metabolites involved with lipid metabolism was markedly different between the moderate and severe trials. Specifically, concentrations of MCFA and LCFAs (stearic acid, arachidic acid, arachidonic acid, and docosatetraenoic acid) increased after the moderate domain trial, whereas LCFAs decreased or remained unchanged after the severe domain trial. Finally, as expected, the log_2_fold change in TCA intermediates, lactate, pyruvate, and alanine was higher in the severe compared to the moderate and heavy domain trials, reflecting the higher glycolytic flux rate associated with exercise performed above the metabolic steady state (i.e., critical speed).

### 4.1 Plasma lactate, pyruvate, and alanine concentration increases immediately after exercise performed in the severe intensity domain

Higher concentrations of lactate, pyruvate, and alanine were measured after the swimming trials performed in the severe compared to heavy and moderate intensity domains. This metabolic response reflects an increased reliance on the TCA and glucose‐alanine cycles for energy provision during exercise performed within the severe intensity domain, which is above the metabolic steady state. Approximately 70%–80% of the lactate produced during high‐intensity exercise is cleared via the skeletal muscle and cell‐to‐cell lactate shuttles after exercise, which helps to replenish skeletal muscle glycogen content (Brooks, 2020). Lactate clearance rates after exercise are affected by athletes' skeletal muscle fiber type (lactate clearance is faster in oxidative fibers), mitochondrial content, and the intensity of active recovery from exercise (as a greater muscle mass is active to promote lactate clearance) (Bergman et al., 1999; Donovan & Brooks, 1983).

While plasma lactate and pyruvate concentrations are elevated in venous blood after exercise, other TCA pathway intermediates can remain trapped in cells, with varying degrees of “spill‐over” into the plasma of metabolites residing in skeletal muscle and liver pools. Lactate acts not only as an important energy substrate both during and after exercise but also dictates the partitioning of energy substrate use during exercise by inhibiting lipolysis and mitochondrial FFA oxidation, with emerging roles in cell redox homeostasis, ROS production, and histone lactylation (Brooks et al., 2022). To some extent, the inhibitory effect of lactate on lipolysis associated with high blood lactate concentrations achieved during the severe intensity domain trial may explain the lower overall FFAs concentration measured in this trial compared to the moderate and heavy domains trials.

Increases in lactate, pyruvate, and alanine similar to those observed in this study have been observed in other studies profiling the plasma metabolite responses to exercise immediately and 1 h after exercise performed in the severe intensity domain using a metabolomics approach (Aird et al., 2022; Danaher et al., 2016; Peake et al., 2014; San‐Millán et al., 2020). Plasma pyruvate, lactate, and alanine increased 1 h after 4–6 × 30 s all out sprints on a bicycle ergometer in untrained males, regardless of whether exercising in a fasted state or having consumed a carbohydrate‐rich drink (0.91 g/kg CHO) (Aird et al., 2022). Additionally, whole blood concentrations of TCA intermediates such as succinate, citrate, fumarate, aconitate, and maleate increased in world‐class cyclists 1 h after they performed a graded exercise test to their limit of tolerance (Aird et al., 2022). In contrast to (San‐Millán et al., 2020), our analysis focused on providing more coverage of metabolites involved in lipid metabolism, meaning we cannot provide further information about the changes in more TCA intermediates after each swimming trial performed in the various intensity domains.

### 4.2 Plasma FFA appearance is greater after exercise performed in the moderate and heavy domains than in the severe domain

We measured a greater increase in plasma lipolysis pathway metabolites (TAGS, FFAs, and VLDLs) approximately 15 min after exercise performed in the moderate and heavy domains compared to the severe domain. The changes in lipolysis pathway intermediates (TAGs, FFAs, large lipoproteins in VLDLs) observed here are consistent with the metabolic phenotype reported elsewhere in plasma, serum, and whole blood after prolonged exercise within the moderate and heavy intensity domains (Manaf et al., 2018; Nemkov et al., 2023; Peake et al., 2014; Stander et al., 2018, 2020), but with some differences in the exact FFA perturbed by exercise. The post‐exercise recovery kinetics of lipolysis pathway intermediates can be separated into immediate (<1 h), early (1–4 h), and late recovery phases (>4 h) (Lundsgaard et al., 2020). Lipolysis pathway metabolite appearance within the plasma immediately after exercise in the moderate and heavy intensity domains is expected as partial (or incomplete) FA oxidation in the skeletal muscle during low‐intensity exercise increases the plasma concentrations of long and medium chain and acylcarnitines and their associated fatty acids (Lehmann et al., 2010).

Exercise duration, intensity, and sex appear to influence the composition of FFAs after exercise (Nieman & Mitmesser, 2017; Peake et al., 2014). In the current study, the plasma concentration of all FFAs identified increased after exercise in the moderate and heavy domains, with the highest log_2_ fold changes measured for myristic acid (FFA 14:0), myristoleic acid (FFA 14:1), n‐3‐alpha‐linolenic acid (FFA 18:3), palmitoleic acid (FFA 16:1), and linoleic acid (FFA 18:2). In comparison, stearic acid (FFA 18:0), arachidic acid (FFA 20:0), n‐6‐arachidonic acid (FFA 20:4), and n‐6‐docosatetraenoic acid (FFA 22:4) showed the largest differences between exercise in the moderate and severe domains. There were few changes in plasma FFA concentration from pre‐exercise values in the severe domain, potentially owing to the inhibitory effect of lactate on lipolysis (Boyd III et al., 1974). Increases in plasma concentrations of myristic acid and palmitoleic acid have been reported elsewhere after prolonged moderate intensity cycling (~67% V̇O_2peak_) (Peake et al., 2014). Prolonged moderate intensity running (~2.26 h at 70% V̇O_2peak_) can increase the concentration of myristic acid, myristoleic acid, palmitoleic acid, linoleic acid, and arachidic acid in the plasma immediately after exercise (Nieman & Mitmesser, 2017). In contrast to our findings, myristoleic acid was unchanged from pre‐exercise values after moderate‐intensity exercise in well‐trained male cyclists and triathletes (Peake et al., 2014). While changes in SCFAs have been detailed after exercise performed in the moderate (Nieman & Mitmesser, 2017; Peake et al., 2014) and severe intensity domains (Danaher et al., 2016; Peake et al., 2014), we were unfortunately unable to compare the MCFA and SCFA concentrations between swimming trials performed in different exercise intensity domains as our analysis method did not detect these metabolites.

### 4.3 Plasma FFA appearance may be lower in men compared to women after exercise in the severe domain

Plasma FFA concentration was lower than baseline or unchanged after exercise performed in the severe intensity domain potentially due to the inhibitory effect of lactate on lipolysis and FFA oxidation when sex was not considered as a covariate. In contrast to our findings, MCFA and LCFA concentrations have been observed to increase in plasma immediately after exercise performed in the severe intensity domain, predominantly in males. In male world‐class cyclists, oleic acid, linolenic acid, docosapentaenoic acid, and docosahexaenoic acid showed a ~1.0–2.5‐fold increase from pre‐exercise values immediately after a graded exercise test (San‐Millán et al., 2020). Similarly, high‐intensity interval training (10 × 4 min at ~81% V̇O_2peak_/2 min active recovery at 50 W) in trained male cyclists and triathletes increased myristic acid, dodecanoic acid, decanoic acid, palmitoleic acid, myristoleic acid, and oleic acid immediately after exercise. In a study of seven untrained males, dodecanoic acid, hexadecanoic acid, and octadecanoic acid increased from baseline 1 h after 30 × 20 s at 150% V̇O_2peak_/40 s passive rest and 10 × 30 s/50 s at 300% V̇O_2peak_/50 s passive rest (Danaher et al., 2016). To some extent, sample collection time points (e.g., immediately post‐exercise vs. 1‐h post‐exercise), exercise intensity, and athletes' training status may partially explain the disparate post‐exercise FFA responses observed in the current study.

**FIGURE 5:**
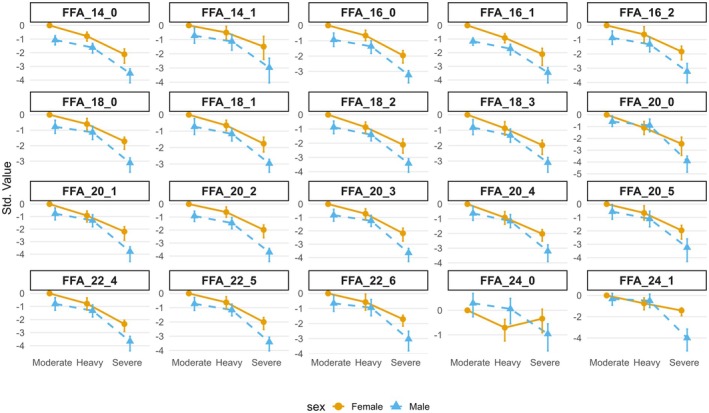
Log_2_ fold change in plasma free fatty acid (FFA) concentration 1 h after swimming exercise performed within the moderate, heavy, severe intensity domains or males (blue triangle) and females (orange circle). Responses have been standardized to show deviation from the moderate intensity domain trial. We note that the log_2_ fold change in FFAs after exercise in the severe domain is lower in males than females for all FFAs.

When the FFA response to exercise was categorized by sex in the current study, visual inspection of plasma FFA log_2_ fold changes from pre‐exercise values indicates the FFA responses occurred in the same direction between males and females in the moderate and heavy intensity domains but were lower in male swimmers (Figure 5). In the severe intensity domain, FFA appeared to decrease from pre‐exercise values in males but increased in females. We used OPLS‐DA to conduct a multivariate examination of the disparate FFA responses between sexes in the severe domain. Unfortunately, we could not statistically distinguish a sex‐specific FFA response, likely owing to our low sample size for this comparison. However, given the exploratory nature of this research, we consider this observation warrants further discussion. Females are thought to rely more on lipid metabolism than males during exercise within the moderate and heavy domains, likely due to higher plasma FA availability and transport capabilities as estradiol 17b promotes lipid over carbohydrate oxidation. However, the evidence for sex differences in energy substrate metabolism is mixed (Sanchez et al., 2024). To some extent, sex differences in FA metabolism seen during high‐intensity exercise may result from the higher muscle mass and lower adipose tissue in male compared to female athletes. In untrained females, ^2^H_2_ palmitate isotope monitoring demonstrated that plasma FA appearance was higher during exercise performed at 25% compared to 85% V̇O_2peak_ (Romijn et al., 2000). However, no difference was observed in plasma FA appearance between males and females when correcting appearance rates for lean body mass (Romijn et al., 2000).

Outside of military settings, few studies have used metabolomics to investigate the differences in metabolic responses between highly trained males and females after high‐intensity exercise. An investigation of the serum metabolome in male and female military personnel completing Cadet Leader Development Training (a physically demanding 17‐day military training course) revealed a greater abundance of metabolites related to fat metabolism (i.e., FAs, endocannabinoids, lysophospholipids, phosphatidylcholines, and phosphatidylethanolamines) in the serum of women soldiers than in men. Hence, under caloric stress, women preferentially mobilize fat compared to men, which could be a way of mitigating losses in lean muscle mass observed in men (Beckner et al., 2023). While we cannot rule out the effect of sex steroid hormones on FFA differences between males and females, the overall exercise‐related metabolome response to exercise is likely more similar than different between males and females. It appears that exercise duration, intensity, the timing and composition of the pre‐exercise meal likely have a greater impact on the plasma FFA responses to exercise than sex steroid hormones. Future research should investigate short‐ and long‐term sex‐specific differences in the metabolomic responses to exercise in the context of female sex steroid hormone cycles and the influence of hormonal contraceptives on the metabolic responses to exercise. Such research may inform sex‐specific nutrition guidelines in well‐trained female athletes.

### 4.4 Study limitations

We acknowledge several limitations in this study, which are the result of trying to maintain ecological validity and the practicalities of working with high‐performance athletes. Firstly, we limited data collection to two squads of high‐performance male and female swimmers to reduce potential between‐squad variation arising from the training programmes employed by different swimming coaches. Secondly, swimmers completed more volume in the heavy compared to the moderate intensity domain (4800 m vs. 2400 m). The difference in training volume was necessary to ensure the swimming sessions remained ecologically valid and fit with the coaches' requirements and swimmers' typical training schedule. Thirdly, feeding status is known to influence energy substrate use during and after exercise. To maintain ecological validity, we did not collect blood samples in a fasted state as our swimming cohort would typically have consumed a light breakfast before their morning exercise. Instead, athletes were asked to replicate the same breakfast before each exercise trial. The molecular profile of pre‐ and post‐exercise samples collected during each exercise trial is therefore influenced both by exercise and the composition of the athletes' pre‐exercise meal. Fourthly, we collected capillary whole blood from the finger within 15 min after exercise. Capillary blood sampling is more practical in an athletic population as it is less invasive than a venous blood sample or skeletal muscle biopsy and is less affected by changes in hydration status after exercise than urine. However, we acknowledge that the abundances of various metabolite classes can differ between whole blood, plasma, serum, urine, and skeletal muscle samples (Thachil et al., 2024).

Different interval prescription methods likely stimulate different metabolic phenotypes for each athlete or specific athlete grouping (i.e., endurance vs. sprint specialists). For example, swimmers focusing on sprint events (50 m, 100 m) may show a different metabolic response to interval trials performed within the severe intensity domain than middle‐distance swimmers (200–800 m) by virtue of disparate long‐term physiological adaptations to training. Future research is necessary to understand how factors such as training history and muscle fiber type influence metabolite responses to acute exercise and long‐term training. We were also limited in our physiological scope to robustly detect lipids, lipoproteins, small molecules, and amino acids. Therefore, we were unable to detect TCA intermediates, SCFA, and MCFA and metabolites involved in purine metabolism. Given the importance of these metabolites during exercise in the severe domain, we recommend including these metabolites in future targeted assays when investigating the athlete metabolome. Finally, our study does not provide insights into the production and clearance of metabolites during or after exercise (i.e., metabolic flux). Conceivably, training‐induced differences in ATP turnover rates during exercise may help explain the between‐athlete variability in performance, while the post‐exercise kinetics of anti‐inflammatory and antioxidant pathways may contribute to between‐athlete differences in post‐exercise recovery. Although future studies using stable isotopes (e.g., ^14^C and palmitate) to compare metabolic fluxes will provide a comprehensive understanding of substrate fluxes (Wilkinson et al., 2017), these studies are currently limited to a laboratory setting and less suited to field‐based collection.

### 4.5 Future directions

Additional research is required to refine our understanding of the molecular responses to exercise and training in high‐performance athletes. Understanding the within‐day and between‐day variability in metabolite responses to exercise of different intensities is necessary to determine the stability and reproducibility of resting and post‐exercise blood metabolite profiles. Some work has been conducted in this area to date (Darragh et al., 2023; Mullen et al., 2020; Sato et al., 2018) and future investigations should have both training and anti‐doping applications. Moreover, exercise training involves weeks to years of repeated metabolic perturbations to develop structural and functional changes. Therefore, further work is required to profile the long‐term post‐exercise metabolic responses to training within exercise intensity domains in high‐performance swimmers. Such research would help better guide exercise prescription to a swimmer's critical speed and race pace, which may yield better metabolic outcomes. Lipid metabolism is lower after exercise performed in a fed versus fasted state. Consequently, a better understanding of how meal timing and nutrient composition affect plasma metabolite profiles after different types of exercise or when exercise is performed at different times of the day (e.g., morning exercise compared to evening exercise, twice daily exercise sessions) may yield new insights. This work will inform the interpretation of changes in specific lipolysis intermediates following exercise performed within the moderate to severe exercise intensity domains.

Few studies have compared the metabolomic profiles of well‐trained male and female athletes in response to exercise across intensity domains, nor have they investigated potential differences in energy substrate metabolism for various sex‐steroid profiles in female athletes. Finally, integrating information from additional omic layers (e.g., transcriptome, proteome, and gut microbiome) and from different tissues (e.g., skeletal muscle and adipose tissue) and biofluids such as urine and saliva will provide a more holistic understanding of the molecular responses to exercise performed in different intensity domains in high‐performance athletes in the future.

## 5 CONCLUSION

In our cohort of high‐performance male and female swimmers, the concentration of metabolites involved in lipid metabolism (FFAs, TAGS, and VLDLs) was higher in the blood plasma immediately after the exercise performed in the moderate compared to heavy and severe intensity domains. In comparison, the concentrations of metabolites involved in the TCA and glucose‐alanine cycles, such as lactate, pyruvate, and alanine, respectively, were substantially higher after exercise performed in the severe intensity domain compared to the moderate domain. Our results demonstrate that exercise performed in different intensity domains exhibits distinct metabolic signatures, which can be detected effectively from a 1.0 mL capillary blood (plasma) sample, therefore reducing the volume of blood required for long‐term metabolomic analysis in athletes.

## AUTHOR CONTRIBUTIONS

AG, LM, NL, CG, DP, and KM designed the study. AG, LM, NL, CG, and KM collected the data. NL conducted the metabolic phenotyping. AG and NL performed the bioinformatics for metabolomics data. AG, LM, NL, CG, DP, MK, and KM wrote the manuscript and approved the final draft.

## Supporting information


Appendix S1.
