# Supplementary material for: Acute metabolite responses to swimming exercise of different intensities in highly trained male and female swimmers
Source: Physiol Rep. 2025 Aug 26;13(16):e70532. doi: 10.14814/phy2.70532 (PMC12381355; doi:10.14814/phy2.70532)
Supplement: Supplementary file 1 — Appendix S1. [file PHY2-13-e70532-s001.docx]

**Supplementary Materials**

**Methods**

UpSet plots were created using the UpSetR package (Conway et al., 2017) in the R statistical programming language. UpSet plots visualise the metabolites altered by each exercise trial and present intersections between multiple sets (i.e., moderate, heavy, and severe intensity domain trials). This approach comprised three main components: the intersection matrix, the intersection size bar plot, and the set size bar plot. The intersection matrix uses columns to represent unique combinations of sets, with filled dots indicating the presence of a set in a specific combination. The intersection size bar plot, positioned above the matrix, illustrates the size of each intersection, with bar heights corresponding to the number of elements shared among the sets. The set size bar plot, located to the left of the matrix, shows the total number of elements in each individual set. This layout facilitates efficient comparisons of complex intersections.

**References**

Conway JR, Lex A & Gehlenborg N. (2017). UpSetR: an R package for the visualization of intersecting sets and their properties. *Bioinformatics* 33, 2938-2940.

**
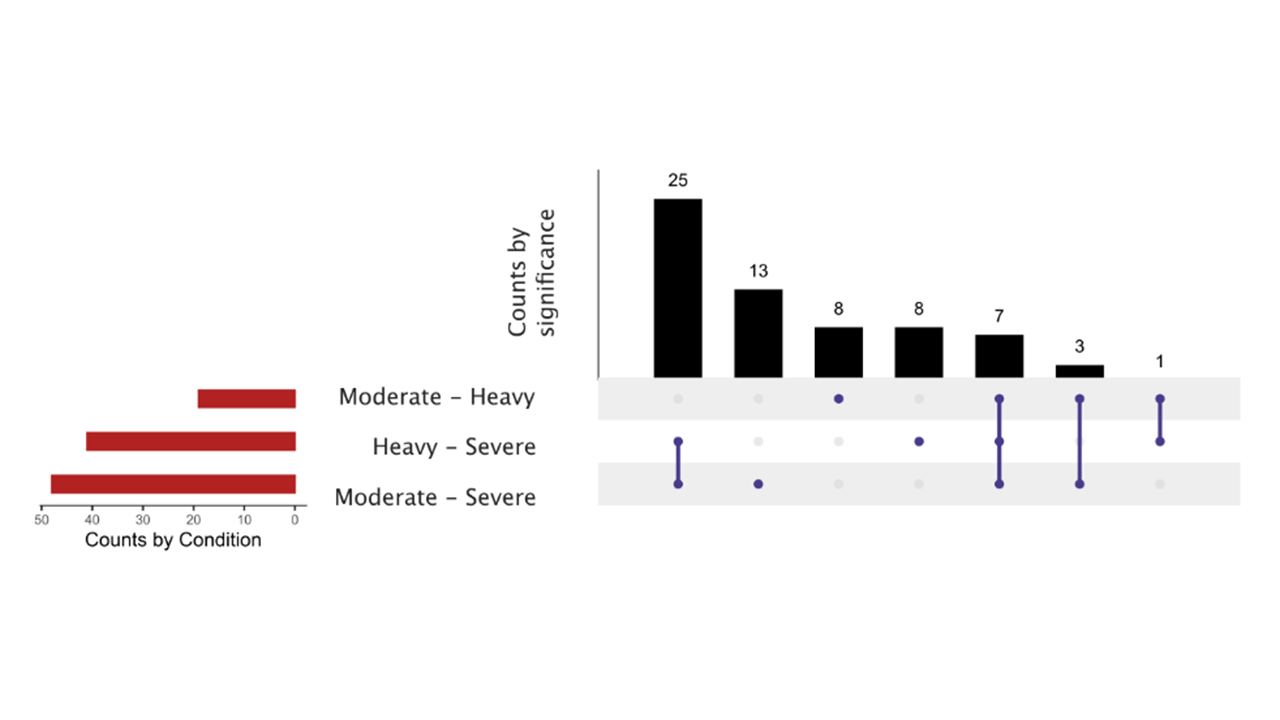
**

**Figure S1:** UpSet plot of the metabolites that were significant in the moderate, heavy, and severe intensity domain trials. The number displayed on each bar represents the total number of metabolites in each corresponding set intersection. For example, in set 5, seven metabolites intersected (i.e., were common) between the three paired trial contrasts (differences) listed. In comparison, set 6 comprises three metabolites overlapping the differences between the moderate and heavy intensity domain trials.

**Table S1:** Metabolites identified in each set taken from the UpSet plot (Figure S1), containing low molecular weight metabolites, lipoproteins, and lipids from the NMR and small molecules measured via mass spectrometry.

| **Set 1** | **Set 2** | **Set 3** | **Set 4** | **Set 5** | **Set 6** | **Set 7** |
| --- | --- | --- | --- | --- | --- | --- |
| Triglycerides, total | Alanine | DAG (16:1, 18:0) | Triglycerides, LDL-4 | FFA (18:2) | Pyruvic acid | PS (16:0, 18:1) |
| LD1, particle number | Apo-B100/Apo-A1 | PE-O-(18:0, 18:0) | Phospholipids, HDL-1 | FFA (18:3) | PE (16:0, 16:0) |  |
| Triglycerides, LDL | Triglycerides, VLDL-5 | PG (18:2, 20:4) | DAG (16:0, 16:0) | FFA (20:1) | PE (14:0, 18:1) |  |
| Triglycerides, HDL | Apo-A2, HDL-3 | TAG (50:0)-FA (16:0) | DAG (16:0, 18:0) | FFA (20:4) |  |  |
| Free cholesterol, VLDL | FFA (24:0) | TAG (50:0)-FA (18:0) | DAG (16:0, 18:1) | FFA (22:4) |  |  |
| Free cholesterol, HDL | LPI (20:2) | TAG (51:0)-FA (16:0) | DAG (18:1, 20:1) | FFA (14:0) |  |  |
| Triglycerides, LDL-1 | PE (16:0, 16:1) | TAG (51:0)-FA (17:0) | PI (18:0, 14:0) | FFA (16:1) |  |  |
| Free cholesterol, LDL-1 | PE (18:0, 16:0) | TAG (52:0)-FA (18:0) | TAG (58:7)-FA (20:4) |  |  |  |
| Apo-B, LDL-1 | PE (18:0, 20:5) |  |  |  |  |  |
| Triglycerides, HDL-1 | PE (18:2, 20:3) |  |  |  |  |  |
| Triglycerides, HDL-2 | TAG (50:6)-FA (20:4) |  |  |  |  |  |
| Cholesterol, HDL-4 | TAG (56:8)-FA (20:4) |  |  |  |  |  |
| Apo-A2, HDL-4 | TAG (56:9)-FA (20:5) |  |  |  |  |  |

**Table S1 (continued):**

| **Set 1** | **Set 2** | **Set 3** | **Set 4** | **Set 5** | **Set 6** | **Set 7** |
| --- | --- | --- | --- | --- | --- | --- |
| FFA (18:0) |  |  |  |  |  |  |
| FFA (18:1) |  |  |  |  |  |  |
| FFA (20:0) |  |  |  |  |  |  |
| FFA (20:2) |  |  |  |  |  |  |
| FFA (20:3) |  |  |  |  |  |  |
| FFA (20:5) |  |  |  |  |  |  |
| FFA (22:5) |  |  |  |  |  |  |
| FFA (22:6) |  |  |  |  |  |  |
| FFA (24:1) |  |  |  |  |  |  |
| FFA (14:1) |  |  |  |  |  |  |
| FFA (16:0) |  |  |  |  |  |  |
| FFA (16:2) |  |  |  |  |  |  |

Apo: Apolipoprotein, FFA: Free fatty acid; LD: HDL: High density lipoprotein, Low density; LDL: Low density lipoprotein; LPI: Lysophosphatidylinositol; PE: Phosphatidylethanolamines, TAG: Triacylglycerol, VLDL: Very low density lipoprotein.
